# Evaluation of clinical factors predicting dysphagia in patients with traumatic and non-traumatic cervical spinal cord injury: a retrospective study

**DOI:** 10.3389/fneur.2024.1376171

**Published:** 2024-05-15

**Authors:** Jin-Woo Choi, Dae Yeong Kim, Sun Young Joo, Donghwi Park, Min Cheol Chang

**Affiliations:** ^1^Department of Physical Medicine and Rehabilitation, Ulsan University Hospital, University of Ulsan College of Medicine, Ulsan, Republic of Korea; ^2^Department of Rehabilitation Medicine, Daegu Fatima Hospital, Daegu, Republic of Korea; ^3^Department of Rehabilitation Medicine, Yeungnam University Hospital, Daegu, Republic of Korea

**Keywords:** deglutition, deglutition disorders, spinal cord injuries, vital capacity, cervical spine, dysphagia

## Abstract

**Introduction:**

Dysphagia is a common complication in patients with cervical spinal cord injury (C-SCI) and can cause various pulmonary complications, such as aspiration pneumonia and mechanical airway obstruction increasing mortality and morbidity. This study evaluated the clinical factors that predict dysphagia in patients with traumatic and non-traumatic C-SCI.

**Methods:**

Ninety-eight patients with C-SCI were retrospectively enrolled in this study and were divided into those with and without dysphagia. Clinical factors such as age, sex, tracheostomy, spinal cord independence measure, pulmonary function test (PFT) including forced vital capacity (FVC), forced expiratory volume in 1 s (FEV1) and FVC/FEV1, American Spinal Cord Injury Association score, Berg Balance Scale, and surgical approach were investigated retrospectively.

**Results:**

Multivariate logistic regression analysis revealed that FVC and the presence of tracheostomy were significantly correlated with dysphagia in patients with C-SCI (*p* < 0.05). FVC and the presence of tracheostomy are useful tools for detecting dysphagia in patients with C-SCI.

**Conclusion:**

Considering the results of our study, early PFTs, especially FVC, in patients with C-SCI and early initiation of dysphagia management and treatment in patients with C-SCI and tracheostomy will be advantageous in lowering the mortality and morbidity due to pulmonary aspiration in these patients.

## 1 Introduction

Cervical spinal cord injuries (C-SCI) are the most severe SCIs, often resulting in complete or partial loss of sensorimotor functions or even fatalities. C-SCIs are divided into traumatic and non-traumatic types (1).

Dysphagia is a swallowing function disorder characterized by the abnormal movement of liquids or a food bolus (2). In patients with C-SCI, the prevalence of dysphagia varies from 16 to 80% (3, 4). Dysphagia after a C-SCI can increase the risk of aspiration pneumonia, leading to increased mortality and morbidity (3, 4).

Complications secondary to dysphagia among patients with C-SCI include pulmonary sequelae, such as transient hypoxemia, chemical pneumonitis, mechanical airway obstruction, bronchospasm, and pneumonia (5, 6). Furthermore, dysphagia is a predisposing condition for aspiration, which may contribute to the development of pneumonia (7); moreover, complications associated with respiration are considered the leading causes of morbidity and mortality in patients with SCI (8, 9). However, despite being associated with significant morbidity, dysphagia remains under-recognized in SCI (5). Therefore, early identification of risk of dysphagia in individuals with C-SCI can prevent or reduce the development of associated adverse complications by facilitating timely diagnosis and treatment (10, 11). Furthermore, awareness of the factors associated with swallowing dysfunction among individuals with C-SCI is crucial and has important implications for medical management strategies in these patients.

Several studies have investigated risk factors for dysphagia in patients with traumatic C-SCI (10), where age, tracheostomy, injury level, severe paralysis, voice quality, peak cough flow, forced expiratory volume in 1 s (FEV1), and anterior cervical surgery were identified as risk factors for dysphagia (3, 10). However, few studies have involved patients with non-traumatic C-SCI. Therefore, this study aimed to investigate the risk factors for dysphagia in patients with both traumatic and non-traumatic C-SCI.

## 2 Materials and methods

### 2.1 Study design and population

This retrospective study was approved by the Institutional Review Board (IRB) of Ulsan University Hospital (IRB number: 22–04-037) and was performed per the Declaration of Helsinki for human experiments. The IRB of Ulsan University Hospital waived the need for informed consent due to the retrospective nature of the study. We retrospectively obtained clinical data such as sex, age, tracheostomy, spinal cord independence measure (SCIM) scores, completeness of SCI, presence of tracheostomy, neurological level, pulmonary function test (PFT) including forced vital capacity (FVC), FEV1, the ratio of FVC to FEV1 (FVC/FEV1), American Spinal Cord Injury Association (ASIA) scores, Berg Balance Scale (BBS) scores, and surgical method from patients with C-SCI at the university hospital between January 2018 and August 2022.

The inclusion criteria were as follows: (i) aged between 20 and 89 years; (ii) hospitalization for the treatment of C-SCI; (iii) weakness or sensory disturbance in both upper and lower extremities due to C-SCI; and (iv) a clear diagnosis of cervical myelopathy (or injury) using magnetic resonance imaging (MRI; C-SCI was defined as a high signal intensity at the cervical spinal cord in T2-weighted C-SCI MRI consistent with cervical myelopathy as confirmed by certified board radiologic specialists).

The exclusion criteria were as follows: (i) inability to undergo the swallowing test or PFT due to poor consciousness; (ii) dysphagia due to neurologic conditions diagnosed by neurologists, including stroke, traumatic brain injury, anoxic brain injury, brain tumor, motor neuron disease, Parkinson’s disease, or Alzheimer’s disease; (iii) dysphagia resulting from any other surgical or medical history, such as laryngeal or tongue cancer or vocal cord paralysis; and (iv) an interval of >28 days between the swallowing test and PFT.

### 2.2 Dysphagia

Patients with C-SCI were divided into dysphagia and non-dysphagia groups based on their symptoms by a rehabilitation medicine specialist (DP) with >10 years of experience in dysphagia treatment. Based on the judgment of the rehabilitation medicine specialist, a patient was assigned to the dysphagia group following positive assessments in bedside swallowing evaluations or at least one patient-reported symptom of dysphagia, such as food pressure, coughing when eating, nausea, or changes in diet. A video fluoroscopic swallowing study (VFSS) was performed on all patients with C-SCI in the dysphagia group to confirm the dysphagia. To evaluate the severity of dysphagia, penetration-aspiration scale (PAS) was used.

### 2.3 The spinal cord independence measure (SCIM)

The SCIM is a scale used to assess the achievement of daily function in patients with spinal cord lesions. The third version (SCIM III) contains 19 tasks organized into three subscales: self-care, respiration and sphincter management, and mobility (12). Here, we compared the effects of dysphagia in patients with C-SCI using scores of two subscales—respiration and sphincter management and mobility—and total SCIM scores. The SCIM was evaluated through observation and interviews with the patient’s occupational therapists.

### 2.4 Pulmonary function test (PFT)

Spirometry was conducted according to American Thoracic Society guidelines (Vmax 22, SensorMedics; PFDX, MedGraphics, Dersingham, United Kingdom). The following parameters were evaluated: FVC, FEV1, and FEV1/FVC (13). All spirometric values are expressed as percentages of the predicted values (13).

### 2.5 American Spinal Cord Injury Association (ASIA)

The ASIA International Standards for Neurological Classification of Spinal Cord Injury form was used to evaluate spinal cord injuries (Atlanta, GA, Revised 2011, Updated 2015. Published with permission of the ASIA, Richmond, VA, United States) (14, 15). The sensory examination evaluates 28 specific dermatomes bilaterally for light touch (generally with a piece of cotton) and pinprick (generally with a clean safety pin) sensations. Each examination component was recorded for each dermatome and laterality. Grades of 0, 1, and 2 denote a lack of sensation, an impaired or altered sensation, and a normal sensation, respectively. A normal unilateral sensory examination comprises 28 dermatomes, each with 2 points for a light touch and 2 points for a pinprick, yielding a total of 112 points. A total score of 224 bilaterally was considered a fully normal sensory examination (14, 15). The motor examination graded five specific muscle groups in the upper and lower extremities, representing major cervical and lumbar myotomes. Motor strength was graded using a universal six-point scale (0–5). Motor strength was recorded bilaterally in each muscle group. The maximum bilateral motor score in a healthy individual is 100, with 50 for scoring 5/5 in all right upper and lower extremity myotomes and another 50 in the left (14, 15).

### 2.6 The Berg balance scale (BBS)

The BBS is a 14-item scale that quantitatively assesses balance and risk of falls in older community-dwelling adults through direct observation of their performance (16). The items were scored from 0 to 4, with a score of 0 representing an inability to complete the task and a score of 4 representing independent completion of the item (16). A physical therapist measured the BBS scores of the patients with C-SCI and calculated the global score from 56 possible points.

### 2.7 Surgical method

Information about the surgical approach—anterior, posterior, or combined anterior–posterior in cervical spine surgery in patients with C-SCI was noted (10).

### 2.8 Statistical analysis

For comparisons of the patient characteristics (age, sex, SCIM scores, ASIA scores, FVC, FEV1, FVC/FEV1 ratio, BBS, and cervical approach) between the two C-SCI groups, *p*-values were calculated using a Pearson chi-square test or Mann–Whitney U-test. Using clinical factors which showed a statistical significance in Pearson chi-square or Mann–Whitney U-tests, multivariate logistic regression analysis using the stepwise method was performed to evaluate the correlation with the presence of dysphagia. We also performed a receiver operating characteristic (ROC) analysis to investigate the sensitivity, specificity, and cutoff value of clinical factors for predicting dysphagia in patients with C-SCI. Statistical analyses were performed using MedCalc (MedCalc Software Ltd., Ostend, Belgium) and SPSS software (version 22.0; IBM Corp., Armonk, NY, United States).

## 3 Results

### 3.1 Patient characteristics

Of the 98 patients with C-SCI enrolled in this study, 72 were male and 26 were female (mean age 64.59 ± 12.53 years). The demographic data of the patients is shown in Table 1. Classification of the causes of C-SCI is presented in Table 2. Among the patients, 34 were classified into the dysphagia group and 64 into the non-dysphagia group. All patients, except for two with transverse myelitis, underwent surgery. The average interval between the VFSS and PFT in our study was 2.22 ± 1.00 days (Min–Max; 1–5 days).

**Table 1:** Characteristics of included patients with cervical spinal cord injury.

Characteristic	Dysphagia (*n* = 34)	Non-dysphagia (*n* = 64)	*p*-value
Age	66.76 ± 13.09	63.44 ± 12.28	0.382
Sex			0.504
Male, *n* (%)	26 (76.50)	46 (71.90)	
Female, *n* (%)	8 (23.50)	18 (28.10)	
SCIM			
SCIM_respiration score	21.08 ± 14.78	14.26 ± 11.15	0.152
SCIM_mobility score	8.77 ± 13.01	5.97 ± 8.70	0.407
SCIM_total score	37.38 ± 31.57	29.42 ± 21.20	0.416
ASIA			
ASIA_motor score	53.13 ± 31.28	57.63 ± 21.91	0.623
ASIA_sensory score	69.80 ± 33.01	108.43 ± 193.81	0.450
Pulmonary function test			
FVC (%predicted)	49.93 ± 27.12	66.47 ± 17.88	**0.020** ^*^
FEV1 (%predicted)	57.36 ± 29.65	72.50 ± 18.20	**0.038** ^*^
FEV1/FVC ratio	86.93 ± 8.60	82.17 ± 8.79	0.099
Cause of injury			0.187
Traumatic, *n* (%)	22 (64.70)	30 (46.90)	
Non-traumatic, *n* (%)	12 (35.30)	34 (53.100)	
Operation			0.248
Anterior approach, *n* (%)	12 (35.30)	32 (50)	
Posterior approach, *n* (%)	22 (64.70)	32 (50)	
Tracheostomy			**0.005** ^*^
Positive, *n* (%)	12 (35.30)	2 (3.1)	
Negative, *n* (%)	22 (64.70)	62 (96.9)	
ASIA impairment scale			0.099
A, *n* (%)	8 (23.5)	4 (6.30)	
B, C, and D, *n* (%)	26 (76.5)	60 (93.8)	
PAS	–	3.94 ± 2.98	

^*^Significant difference was noted between the two groups (*p* < 0.05). *p*-value was calculated by comparing dysphagia and non-dysphagia groups using independent *t*-test, Mann–Whitney U-test, or Pearson’s chi-square test. SCIM, spinal cord independence measure; FVC, forced vital capacity; FEV1, forced expiratory volume in one second; PAS, penetration-aspiration score. Bolds indicate *p* < 0.05.

**Table 2:** Classification of causes of cervical spinal cord injury in patients.

Cause	*n* = 98
Traumatic	70 (71.4%)
Non-traumatic	
Transverse myelitis	4 (4.1%)
Ossification of posterior longitudinal ligament	20 (20.5%)
	98 (100%)

### 3.2 The difference in parameters between the dysphagia and non-dysphagia groups

In the independent Pearson chi-square test or Mann–Whitney U-test, FVC, FEV1, and the presence of tracheostomy were significantly different between the dysphagia and non-dysphagia groups (*p* < 0.05; Table 1). There was no significant between-group difference in the age, sex, SCIM score, cause of injury, ASIA motor and sensory score levels, and completeness of SCI (*p* > 0.05; Table 1).

In the multivariate logistic regression analysis, FVC and the presence of tracheostomy were found to be significantly associated with dysphagia in patients with C-SCI (*p* < 0.05; Table 3).

**Table 3:** Multivariate logistic regression analysis (with the Stepwise method) of the presence of dysphagia and FVC and tracheostomy in the patients with cervical spinal cord injury.

	B	Standard error	*p*-value	Exp(B)	95% CI	
					Lower bound	Upper bound
FVC	−0.007	0.003	**0.017** ^*^	−0.326	−0.012	−0.001
Tracheostomy	0.572	0.178	**0.003** ^*^	0.422	0.213	0.931

CI, confidence interval; FVC, forced vital capacity. ^*^ < 0.05. Bolds indicate *p* < 0.05.

**Figure 1:**
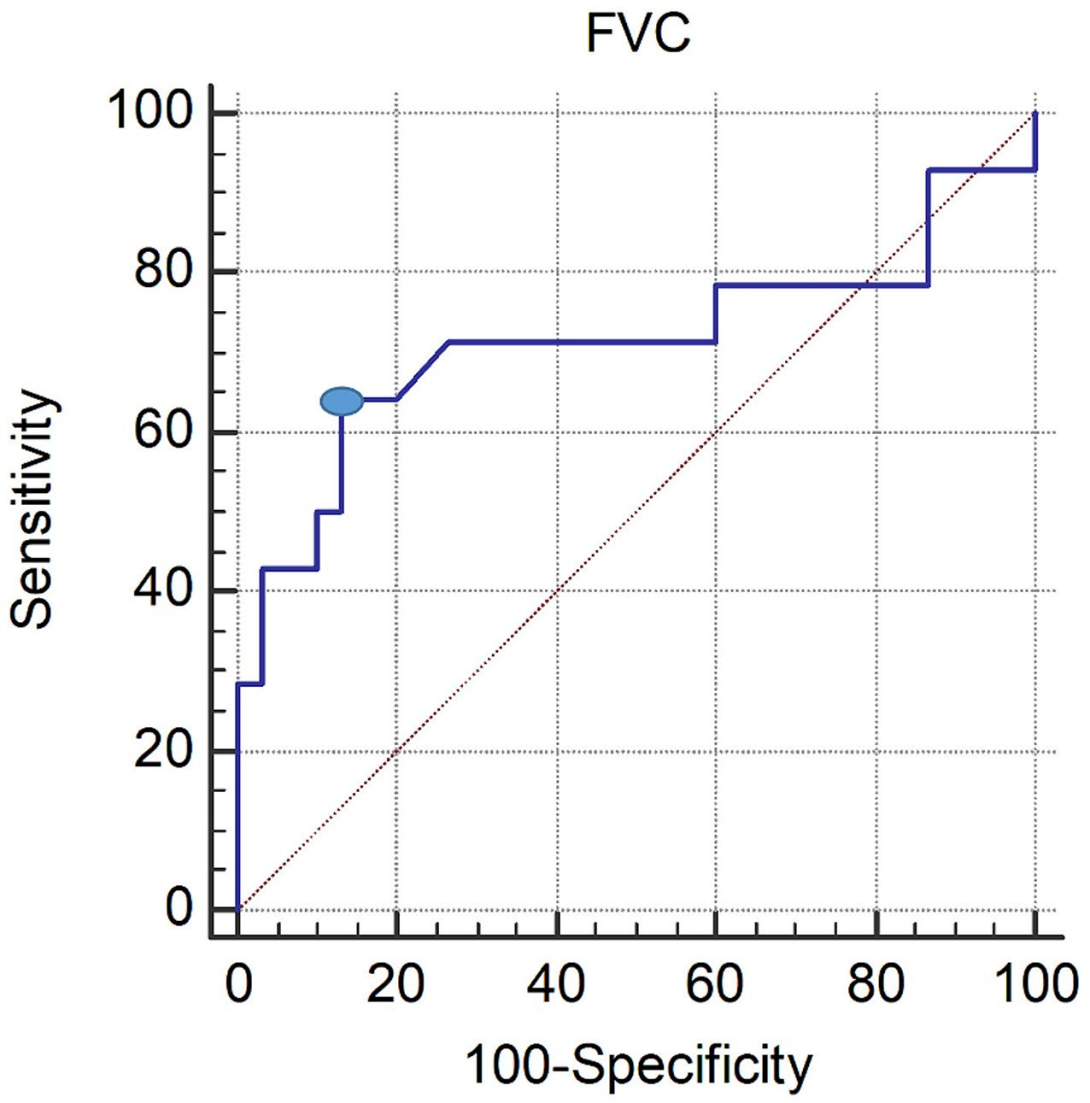
ROC curve of the FVC in patients with dysphagia. The optimal cutoff value (dot on the curve) of the FVC, obtained from the maximal Youden index, was ≤48% predicted (AUC, 0.737; 95% CI, 0.530–0.943; *p* = 0.024; sensitivity 69.23%; specificity 84.21%). ROC, receiver operating characteristic; FVC, forced vital capacity; AUC, area under the ROC curve; CI, confidence interval.

In the ROC curve analysis, the area under the ROC curve of FVC for the presence of dysphagia was 0.714 (95% confidence interval 0.558–0.840; *p* = 0.0386). The optimal threshold for detecting dysphagia in patients with C-SCI was determined to be a predicted maximal Youden index of ≤48% (sensitivity, 64.29%; specificity, 86.67%; see Figure 1).

## 4 Discussion

Several studies investigating the risk factors for dysphagia in patients with traumatic C-SCI have identified age, tracheostomy, severe paralysis, voice quality, PFT results (peak cough flow or FEV1), and anterior cervical surgery as risk factors for dysphagia in patients with traumatic C-SCI (3, 10). To the best of our knowledge, no previous study has investigated the risk factors for dysphagia involving both patients with traumatic and non-traumatic C-SCI. The results of this study suggest that the presence of tracheostomy and FVC ≤48% of predicted are significantly associated with dysphagia (sensitivity, 64.29%; specificity, 86.67%). The reason our study had fewer risk factors for dysphagia compared with previous studies is thought to be the inclusion of both traumatic and non-traumatic C-SCI. The FVC is correlated with the inspiratory muscles’ power (17). Although the inspiratory muscles comprise several muscle groups; the main inspiratory muscles are the diaphragm, which is innervated by the phrenic nerve originating from the C3-C5 root (18). However, there were no statistically significant between-group differences in the SCIM motor and sensory scores, completeness of SCI, or the ASIA motor and sensory scores, indicating the severity of SCI. Considering this, these same spinal pathways (e.g., for intercostals and accessories) are also important in swallowing, and additionally affect swallow-breathing coordination (19). In addition, the relationship between swallowing and breathing may have influenced these results, as precise coordination between the two is important to prevent pulmonary aspiration (20). Although the exact neural processes by which these behaviors (breathing and swallowing) are coordinated are not well understood, it is hypothesized that swallowing and breathing are coordinated to occur at specific times relative to one another to minimize the risk of aspiration by a common control system present in the brainstem (21). In addition, FEV1, which measures the instantaneous expiratory volume for 1 s and was reported to be correlated with dysphagia in traumatic C-SCI, may be affected more by other clinical factors such as airway obstruction than FVC due to its shorter measurement time. Therefore, PFT parameters, especially FVC, which can represent breathing ability and ventilation to some extent, may be associated with the presence of dysphagia in patients with C-SCI.

In addition, we found that the presence of tracheostomy was significantly different between dysphagia and non-dysphagia groups in patients with C-SCI. In many previous studies, tracheostomy has been associated with swallowing difficulty, such as increased incidence of aspiration, and several possible mechanisms have been proposed. These are, first, the reduction of laryngeal elevation due to the tethering of the larynx by the tracheostomy tube (22); second, occlusion of the pharyngeal pathway by the tracheal tube cuff (23); third, loss of protective reflexes and desensitization of the larynx due to chronic air diversion (24); fourth, uncoordinated laryngeal closure due to chronic upper airway bypass (25). Considering this, early management and treatment of dysphagia in patients with C-SCI and tracheostomy will help lower the mortality and morbidity due to pulmonary aspiration.

In our previous study (26), we examined the correlation between various clinical data (age, sex, severity of SCI, PFT results, BBS, and surgical methods) and the severity of dysphagia in 56 patients with traumatic C-SCI distinct from those included in the current study. We found that the anterior surgical approach was the only significant factor associated with dysphagia severity in these patients. In the previous study (26), we recommended that clinicians should pay particular attention to the potential for occurrence of dysphagia in patients who undergo anterior cervical surgery. Unlike our previous study (26), the present study included both traumatic and non-traumatic patients with C-SCI. Furthermore, instead of assessing dysphagia severity, we evaluated the presence or absence of dysphagia. We compared patient characteristics between the traumatic and non-traumatic C-SCI groups. The current study revealed that FVC results and the presence of tracheostomy are associated with the development of dysphagia in patients with C-SCI.

In this study, the interval between PFT and VFSS was set relatively short (1–5 days). Recovery in patients with C-SCI varies individually, and the speed of recovery can be influenced by various factors. Therefore, assessing lung function and swallowing ability over time is crucial in determining accurate diagnosis and treatment direction. Performing these tests at the appropriate time allows for the evaluation of respiratory and nutritional status and the initiation of appropriate treatment. This contributes to the improvement in overall recovery and quality of life for patients.

Our study has a few limitations. First, this was a retrospective review of medical records, which had the inherent limitations of small sample sizes and heterogeneity. Comparison with previous prospective studies (27, 28) suggests that selective bias may not be completely excluded. Second, the number of patients with non-traumatic C-SCI was relatively small, which may be due to differences in prevalence, and further studies with a larger number of patients with non-traumatic C-SCI are necessary. Third, in our study, VFSS was not performed on all patients with C-SCI. Screening patients with dysphagia using the bedside swallowing test and clinical symptoms may be problematic because patients with asymptomatic dysphagia may be missed. However, to avoid unnecessary long-term radiation exposure due to continuous fluoroscopy during VFSS (10 ~ 20 min), in our hospital, we tried to minimize the above limitation by observing the clinical symptoms related to dysphagia daily and performing the bedside swallowing test at the following two time points: at the time of the first consultation with the department of rehabilitation medicine and immediately upon transfer to the department of rehabilitation medicine. At these two points, based on the judgment of the rehabilitation medicine specialist with more than 10 years of experience in the evaluation and management of dysphagia, patients with C-SCI with positive assessments in bedside swallowing evaluations or reports of at least one symptom of dysphagia, such as food pressure, coughing when eating, nausea, or changes in the diet at any time, underwent VFSS. Patients showing penetration or aspiration in VFSS (PAS ≥2) were assigned to the dysphagia group, and the management and treatment were prescribed. To evaluate the clinical factors correlated with dysphagia in traumatic and non-traumatic C-SCI more precisely, however, further prospective studies with routine VFSS in C-SCI with numerous patients with C-SCI may be necessary. Fourth, this study was conducted at a single tertiary hospital, and future multicenter studies could address the potential bias in the findings reported in this study. Therefore, further prospective studies with larger sample sizes are needed for more accurate generalization.

In conclusion, FVC and the presence of tracheostomy are useful tools for detecting dysphagia in patients with C-SCI. Considering the results of our study, early PFTs, especially FVC, in patients with C-SCI and early initiation of dysphagia management and treatment in patients with C-SCI and tracheostomy will greatly benefit in lowering the mortality and morbidity due to pulmonary aspiration.

## Data availability statement

The raw data supporting the conclusions of this article will be made available by the authors, without undue reservation.

## Ethics statement

The studies involving humans were approved by the Institutional Review Board (IRB) of Ulsan University Hospital. The studies were conducted in accordance with the local legislation and institutional requirements. The ethics committee/institutional review board waived the requirement of written informed consent for participation from the participants or the participants' legal guardians/next of kin because retrospective nature of the study.

## Author contributions

J-WC: Conceptualization, Data curation, Formal analysis, Investigation, Methodology, Resources, Validation, Visualization, Writing – original draft, Writing – review & editing. DK: Conceptualization, Data curation, Formal analysis, Investigation, Methodology, Resources, Validation, Visualization, Writing – original draft, Writing – review & editing. SJ: Conceptualization, Data curation, Formal analysis, Investigation, Methodology, Resources, Validation, Visualization, Writing – original draft, Writing – review & editing. DP: Conceptualization, Data curation, Formal analysis, Investigation, Methodology, Resources, Supervision, Validation, Visualization, Writing – original draft, Writing – review & editing. MC: Conceptualization, Data curation, Formal analysis, Investigation, Methodology, Resources, Supervision, Validation, Visualization, Writing – original draft, Writing – review & editing.
